# Supplementary material for: Feasibility of post-exposure-prophylaxis with single-dose rifampicin and identification of high prevalent clusters in villages’ hyperendemic for leprosy in Senegal
Source: PLoS Negl Trop Dis. 2024 Feb 1;18(2):e0011755. doi: 10.1371/journal.pntd.0011755 (PMC10863860; doi:10.1371/journal.pntd.0011755)
Supplement: Attachment — Submitted filename: Point_by_point response to reviewers_last.pdf [file pntd.0011755.s001.pdf]

12/12/2023

Joseph M. Vinetz
Section Editor
PLOS Neglected Tropical Diseases

Dear Joseph Vinetz,

Ref: Submission ID (PNTD-D-23-01341) - [EMID:3bf452032802a97d]

We are thankful for your email dated 22 November 2023 and for your comments/suggestions including those of the reviewers. We have been carefully through the peer review and have revised the manuscript accordingly.
Please find for your kind consideration the following:

1) A "point by point" response to the comments and suggestions of the Editor and Reviewers (below covering pages). The comments of the reviewers are highlighted in bold font and we respond using normal font and bullet points.

2) A new revised version of the manuscript marked 'tracked changes'.

While hoping that these changes will meet with your favorable consideration, we hold ourselves at your entire disposition for any further information or other changes you might require.

On behalf of all co-authors,

Dr. Gilbert Batista
Corresponding author

POINT BY POINT RESPONSE TO THE REVIEWERS SUGGESTIONS

METHODS

Reviewer #1

The methods are fine

- Not applicable, as the reviewer has not addressed specific comments.

Reviewer #2

The objectives of the study are clearly articulated and are well align with the design of the study. The population choosen for the study is appropriate for the implementation of the conducted research. Also the sample size is sufficient enough to ensure the significance of the results. Some terms used in the methodology section are not very clear. When the authors talked about listing or examining the contacts of the leprosy index cases, it was often not clear, are they talking about the family / household contacts or social contacts or neighbours. Each time contacts are mentioned the group should specified.

- In our study we categorized two groups of contacts: household contacts, and social contacts. Household contacts include all household members living in the same household of an index case. Social contacts include all those living in different households than the index cases, we modified line 48 of the abstract for improving the clarity. We assessed then in the spatial analysis the risk of distance of all contacts according to the physical distance to the nearest index case. As such, contacts were included in six categories same household, 1-25m, 26-50m, 51-75m, 76-100m, and those living at more than 100m (see modified lines 61-65).

It would have been helpful for the readers if the method section would cleary mention how many leprosy index cases were taken into consideration when designing the intervention. Also how many contacts per each leprosy index case were planned to be screened.

- When the intervention was designed, we had no clear idea of the number of index leprosy cases per village as this data is not included in the leprosy registers. Only the estimated population was known. As the estimated number of index cases was unknown, we could not determine the number of family contacts.

RESULTS

Reviewer #1

Results presentation is confusing - see comments

- See our response to reviewer 1 in the section " Summary and general comments".

Reviewer #2

The analysis or the data presented is well align with the methodology or the analysis plan. However, there are some inconsistencies in the numbers that mentioned throughout the paper. For example, the authors mentioned at one point that 6,121 persons were examined / screened and another time the number was mentioned as 6,124. In addition to that, if

only 6,121 or 6,124 contacts were examined, how come that 6,554 were eligible and 6,408 were given SDR-PEP.

- In the 9 villages of the intervention, a total of 9086 household members were listed. Among them, 7219(80%) were present at the moment of the survey, from which 7115 (99%) had accepted to be examined (see modified lines 200-210).
- Of the 7115 household members screened, 561 (7.9%) were excluded from the SDR because they were ineligible. A total of 6408 (97.8%) people of the 6554 eligible took a single dose of rifampicin as post-exposure prophylaxis with SDR-PEP (see non-modified lines 225-227).
- A total of 39 new cases were detected in 6 villages. Excluding the population of the three Social Rehabilitation Villages (SRVs) with no new cases (991), among the 7,115 contacts that accepted to be examined we had a total population of 6124. (see corrected line 216).

Furthermore, in the result section, it was also not always clear how the numbers were calculated, as sometimes only absolute numbers or percentages were given. However, these do not add up when recalculating. The number of contacts who were listed is also not consistent throughout the manuscript. The number of household members (9081) listed and the number of contacts who are listed (7850) are a bit confusing.

- We have corrected the erroneous data in the manuscript. There were 9086 household members instead of 9081 (corrected line 200), which is coherent with the uncorrected "Total" figure in the table 1 under the column "Household members listed".

CONCLUSIONS

Reviewer #1

Conclusions are not supported by the data presented. See comments

- See our response to reviewer 1 in the section " Summary and general comments".

Reviewer #2

The conclusion is supported by the data presented. Limitations are also clearly described. The manuscript clearly illustrates the risk of getting leprosy among the household members of the leprosy cases which is very relevant, especially for the low-endemic settings. However, it was not clear why the camp approach if no cases were detected among the household and the risk to other groups is even lower?

- We agree that camp approach might be the best approach when no recent cases were detected. See lines 275 and 276 were we wrote: "Three SRVs had no new cases, a camp approach could be foreseen if no cases are detected in between."

REVIEW

Reviewer #1

Review and copy-editing is needed to eliminate typos and correct sentences that are incomplete/do not make sense.

- We carefully revised the document and eliminated typos and corrected sentences that were unclear.

Reviewer #2

- No comments, therefore not applicable.
-

SUMMARY AND GENERAL COMMENTS

Reviewer #1

Batista and co-authors present in this manuscript the findings of a campaign consisting of active leprosy case detection and post-exposure prophylaxis in Senegalese villages that emerged from former leprosy colonies. They report a high number of new cases with epidemiological characteristics indicating diagnosis delays and recent transmission, and high acceptance of prophylactic treatment. New cases were clustered, yet the authors report no elevated risk beyond the household of index cases, which is counter-intuitive. While this is a relevant study for leprosy control program design, the manuscript suffers from a number of inaccuracies and shortcomings which need to be addressed before it can be considered for acceptance.
- Abstract: the results section is confusing – re-organize with only one topic in one sentence (e.g. the first sentence which reports new cases, screening rates and SDR acceptance)

- The correction was made in the revised manuscript (corrected lines 58-66).

Abstract: The reported number of individuals screened is lower than the reported number of individuals who received SDR. Since screening is a precondition for SDR this is not possible

- The correction was made in the revised manuscript. In the 9 villages of the intervention, a total of 9086 household members were listed. Among them 7219(80%) were present at the moment of the survey among 7115 (99%) accepting to be examined. Of the 7115 household members screened, a total of 6408 (97.8%) people of the 6554 eligible took a single dose of rifampicin as post-exposure prophylaxis (SDR-PEP). 39 new cases were detected in only 6 villages. Excluding the three VRS with no new cases, a population of 6124 was examined among 7,115 examined (lines 58-66 in the abstract section).

Abstract and Results: Conclusion: "Only individuals living in the same household as the leprosy patient had a significant risk of contracting leprosy." This is clearly not correct (see table 4) – rather, people outside the household of the index patient had a risk similar as the general population, which was still high based on the reported figures (note: most new cases were reported outside the index case households!).

- Although the risk of contracting leprosy exists outside households, it is low compared to family contact where the risk was higher and statistically significant [OR 4.18 (1.74 - 10.04)].

Thus, also the statement "All new cases were detected in households where there are former cases (index cases)" is incorrect.

- This sentence has been deleted from the revised manuscript.

Were the leprosy villages created in 1976 or 1979?

- The villages were created in 1976 by law no. 76-03 of 25 March 1976 on the treatment of leprosy and the social rehabilitation of cured and mutilated lepers. We corrected line 116.

Results: the stated number of 9081 household members seems implausible given the overall size of the villages. Also, household members should not be called social contacts. Why were not all contacts listed? The following figures are also odd: 7115 accepting to be examined, 6124 examined, 7850 listed – all these figures do not add up and do not make sense.

- The correction was made in the revised manuscript. In the 9 villages of the intervention, a total of 9086 household members were listed. Among them 7219(80%) were present at the moment of the survey among 7115 (99%) accepting to be examined. Of the 7115 household members screened, a total of 6408 (97.8%) people of the 6554 eligible took a single dose of rifampicin as post-exposure prophylaxis (SDR-PEP). 39 new cases were detected in only 6 villages. Excluding the three VRS with no new cases, a population of 6124 was examined among 7,115 examined. (lines 58-66 in the abstract section).

Check for typos (e.g. intervale -> interval, M. Leprae -> M. leprae, SDR-EP -> SDR-PEP)

- These items has been corrected and harmonised in the revised manuscript.

<u>**Reviewer #2**</u>
The paper is very relevant and the methodology is clearly articulated. This helps other researchers to replicate the study in different settings. The study is also very useful to understand the risk of getting a leprosy infection among the household contacts of the index cases. Please, recheck the numbers used in the methods and results section. Sometimes they do not add up. Having a clear description of the contact groups and numbers would be very helpful.

- These aspects has been corrected in the revised manuscript.

Dr. Gilbert Batista
On behalf of co-authors
